# Effects of IV dexmedetomidine as a pre-medication on clinical profile of bupivacaine spinal anaesthesia in lower abdominal surgeries: a randomized clinical study

**DOI:** 10.11604/pamj.2022.41.74.28865

**Published:** 2022-01-26

**Authors:** Amit Kumar Choudhary, Mukesh Kumar Prasad, Ravi Keshri, Sneha Choudhary

**Affiliations:** 1 Department of Critical Care Medicine, Manipal Hospital, Bengaluru, India,; 2 Department of Anaesthesia, Teerthanker Mahaveer Medical College, Teerthanker Mahaveer University, Moradabad, Uttar Pradesh, India,; 3 Department of Anesthesia, Indira Gandhi Institute of Cardiology, Patna Medical College and Hospital (PMCH) Campus Ashok Raj Path, Patna, Bihar, India,; 4 Department of Oral Medicine and Radiology, Teerthanker Mahaveer Dental College and Research Centre, Moradabad, Uttar Pradesh, India

**Keywords:** Anesthesia, dexmedetomidine, spinal anesthesia

## Abstract

**Introduction:**

to evaluate the effects of intravenous (IV) dexmedetomidine as a pre-medication on clinical profile of bupivacaine spinal anaesthesia in lower abdominal surgeries.

**Methods:**

this prospective randomized double blind study was done on 60 patients with ASA grade I/II undergoing lower abdominal surgeries under bupivacaine spinal anaesthesia. They were allocated to group-1 and group-2. Group-1 (control group) received normal saline and group-2 (study group) received IV dexmedetomidine 1 µg/kg over 10 min as premedication. Five minutes after premedication, subarachnoid block (SAB) was given with 3 ml of 0.5% hyperbaric bupivacaine following which sensory and motor blockade, hemodynamic changes, sedation, and complications of the surgery were recorded and this data was analyzed statistically using χ^2^ test, corrected χ^2^ test, Fisher´s exact test, and test of proportion (Z-test).

**Results:**

the results of the present study showed that in group-2 there was significant decrease in the onset of sensory block, higher level of sensory blockade achieved, less time required to attain highest level of anaesthesia, prolonged time required for 2 dermatomal regression, prolonged duration of sensory blockade, prolonged duration of analgesia, decrease in onset of motor blockade, no significant increase in duration of motor blockade, there was overall hemodynamic stability except for few cases of bradycardia responding to atropine and hypotension responding to mephentramine, adequate and acceptable intraoperative sedation, and significantly less incidence of shivering in perioperative period.

**Conclusion:**

IV infusion of dexmedetomidine 1 µg/kg body weight prior to SAB can be recommended to achieve better sensory blockade and adequate hemodynamic stability and sedation.

## Introduction

Spinal anaesthesia remains one of the basic techniques in modern anaesthesia which is a commonly used technique in anaesthesia practice for gynaecological, lower abdominal, pelvic, and lower limb surgeries. Spinal anaesthesia is advantageous in that it uses a small dose of anaesthesia, simple to perform and offer a rapid onset of action, reliable surgical analgesia and good muscle relaxation. The advantage is sometime offset by relatively short duration of action and complaints of postoperative pain when it wears off [1,2].

Bupivacaine, when used in recommended doses, under spinal anaesthesia produces complete sensory and motor blockade, but if the duration of surgery prolongs it may have to be converted into general anaesthesia or supplemented with an intravenous anaesthetic agent [3]. Dexmedetomidine is a highly selective α-2 adrenergic agonist with an affinity of eight times greater than clonidine (another α-2 adrenergic agonist). It was approved by the Food and Drug Administration (FDA) in 1999 for use in humans for short term sedation in intensive care unit [4,5].

Dexmedetomidine possesses anxiolytic, sedative, analgesic and sympatholytic properties. It might be a useful adjunct for premedication, especially for patients susceptible to preoperative and perioperative stress [6]. Dexmedetomidine potentiates the anesthetic effects of all intraoperative anesthetics, regardless of method of administration [7]. Hence the objective of the present study was to evaluate the effects of intravenous dexmedetomidine as a pre-medication on the duration of subarachnoid block (SAB), hemodynamic changes and sedation in patients undergoing lower abdominal surgeries (upto T-10 Level) under bupivacaine spinal anaesthesia.

## Methods

Sixty patients between the age group of 18-60 years having American society of anaesthesiologists (ASA) physical status I and II scheduled for lower abdominal surgeries with a duration of 30-150 min under spinal anaesthesia were included in the present study. Whereas, patients having ASA grade III and IV, those with a psychologically unstable status with preexisting neurological deficits, patients posted for caesarean section and those having allergy or hypesensitivity to the study drugs were excluded from the study.

**Study design:** the present study was designed as a prospective, randomized double blind study. Approval from the institutional ethical committee was obtained with reference no. TMMC/IEC/2017/023, and written informed consent from each patient was also obtained. The patients were divided into following two groups based on envelop technique: 1) Group 1: 10 ml normal saline (NS) was given intravenously for 10 minutes by infusion pump prior to SAB; 2) group 2: single bolus dose of dexmedetomidine 1 µg/kg body weight intravenously diluted in 10 ml NS was given over 10 minutes by infusion pump prior to SAB.

One day prior to surgery, procedure was explained to all the patients and written informed consent was obtained from each patient. Patients were kept nil per oral overnight before surgery. They were premedicated with tablet pantoprazole 40 mg orally, the night before surgery. IV access was secured using 18 G IV cannula and infusion of 500 ml ringer lactate was started in all the patients included in the study. Patients were infused with drug as per group in operative room for 10 minutes. Under all aseptic precautions lumbar puncture was done with 26 G Quincke´s needle at L3-L4 interspinous space and 0.5% (H) bupivacaine 3 ml (15 mg) was injected over 30 sec and the patients were placed in supine position immediately. Oxygen (O_2_) was given by face mask at the rate of 4 L/min throughout the surgical procedure. Vital parameters like heart rate, blood pressure and SPO_2_ were recorded immediately after the SAB was given and after every 5 min till the end of the surgery and every 15 min after completion of surgery in post anaesthesia care unit (PACU). All the durations were calculated and recorded considering the time of spinal injection as time 0 (T0).

**Sensory blockade:** it was checked by pin prick method in mid-axillary line [8]. Sensory blockade was assessed from T0 to every 2 minutes for first 10 minutes and then every 15 minutes during surgery and postoperatively. Surgery was allowed to start only when a sensory level of T10 was achieved. Observations made after sensory blockade were as follows: time required for loss of pin prick sensation at T-10 level (onset of sensory blockade); maximum sensory level reached and time required for the same; time required for 2 dermatomal regression; time required for regression of sensory level at L1 (duration of sensory block); time for first request of postoperative analgesia (duration of analgesia) was noted (at this point the study was ended).

**Motor blockade:** it was assessed by modified bromage scale. Time taken for motor blockade to reach modified bromage scale 3 (onset of motor blockade) and regression of motor blockade to modified bromage scale 0 (duration of motor blockade) was noted. Motor blockade was assessed from T0 to every 2 min for first 10 min before surgery and after surgery every 15 min in PACU [9].

**Level of sedation:** it was assessed by Ramsay level of sedation scale. The level of sedation were evaluated both intraoperatively and postoperatively every 15 min using Ramsay sedation scale till 30 min postoperatively. Excessive sedation is defined as score greater than 4. Continuous monitoring of blood pressure (BP), heart rate (HR) and SPO_2_ was done till the end of the study. Intraoperative complications like hypotension (systolic blood pressure (SBP) less than 90 mm Hg or more than 30% fall from baseline value), bradycardia (heart rate <50/min) were managed with IV mephentermine 6 mg bolus and IV atropine 0.6 mg respectively and postoperative complications like nausea, vomiting and shivering was noted and treated accordingly. Time for the first request of postoperative analgesia (duration of analgesia) was noted from T0. Data thus collected was tabulated and subjected to statistical analysis and observations and results were made accordingly. Statistical analysis was performed with the help of Epi Info (TM) 3.5. Using this software, basic cross-tabulation and frequency distributions were prepared [10].

## Results

Demographic profile (age, gender, weight, height), ASA physical status (Table 1) and type of surgery (Table 2) in both the groups were comparable.

**Table 1:** comparison of different parameters of sensory blockade in both the groups

Parameters (mean ± s.d.)	Group 1 (n=30)	Group 2 (n=30)	Test statistic (t58)	p-value
Onset of sensory blockade (in min)	5.60 ± 1.22	3.53 ± 1.20	6.62	0.0001
Highest level of sensory block	T7.20 ± 1.63	T5.20 ± 1.45	5.02	0.0001
Time required for attaining highest level of sensory block (in min)	7.60 ± 1.43	6.27 ± 1.36	3.69	0.0005
Time required for 2 dermatomal regression of sensory blockade (in min)	86.50 ± 15.93	119.00 ± 13.61	8.49	0.0001
Duration of sensory blockade (in min)	137.00 ± 14.06	206.33 ± 16.91	17.26	0.0001
Time for first request of post-operative analgesia (duration of analgesia) (in min)	176.50 ± 13.84	288.17 ± 19.32	25.73	0.0001

**Table 2:** comparison of different motor blockade parameters in both the groups

Parameters (mean ± s.d.) (in minute)	Group 1 (n=30)	Group 2 (n=30)	Test statistic (t58)	p-value
Onset of motor blockade	8.60 ± 1.50	7.07 ± 1.36	4.13	0.0001
Duration for motor blockade	123.00 ± 9.43	124.50 ± 14.46	0.47	0.64

**Sensory blockade:** corrected Chi-square test showed that the time required to achieve highest level of sensory blockade, time required for two dermatomal regression and time required for rescue analgesia was higher in group 2 as compared to group 1. The time required to reach maximum sensory level was significantly lower in group-2 in comparison with group-1 (Table 1).

**Motor blockade:** time taken for motor blockade to reach modified bromage scale 3 in group 2 was significantly lower than group 1. However, Chi-square test showed no statistically significant difference between duration of motor blockade in both the groups (Table 2).

**Ramsay sedation score:** mean sedation (SED) of group 1 was significantly lower than that of group 2 at different time intervals intra-operatively, whereas postoperatively there was no statistically significant difference in the mean SED level in both the groups (Table 3).

**Table 3:** comparison of sedation score at different time intervals in both the groups

Sedation score	Group 1 (n=30) (mean ± s.d.)	Group 2 (n=30) (mean ± s.d.)	Test statistic (t58)	p-value
Base line	2.00 ± 0.01	2.01 ± 0.50	0.11	0.91
Intra-operative				
After 15 min	2.00 ± 0.00	3.13 ± 0.43	14.39	0.0001
After 30 min	2.00 ± 0.00	3.30 ± 0.47	15.14	0.0001
After 45 min	2.07 ± 0.45	3.33 ± 0.55	9.71	0.0001
After 60 min	2.53 ± 0.63	3.47 ± 0.57	6.06	0.0001
After 75 min	2.46 ± 0.51	3.40 ± 0.50	7.20	0.0001
After 90 min	2.00 ± 0.00	3.33 ± 0.48	15.17	0.0001
After 105 min	2.00 ± 0.00	3.50 ± 0.52	15.79	0.0001
After 120 min	2.00 ± 0.01	3.00 ± 0.01	7.29	0.0001
Post-operative				
After 0 min	2.00 ± 0.00	2.23 ± 0.43	2.92	0.006
After 15 min	2.00 ± 0.00	2.00 ± 0.00	0.01	0.99
After 30 min	2.00 ± 0.00	2.00 ± 0.00	0.01	0.99

**Figure 1:**
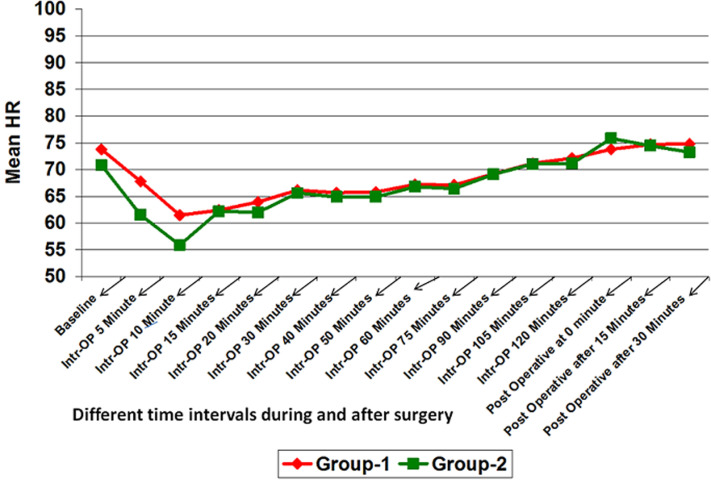
line diagram showing comparison of heart rate at different time intervals in both the groups

**Figure 2:**
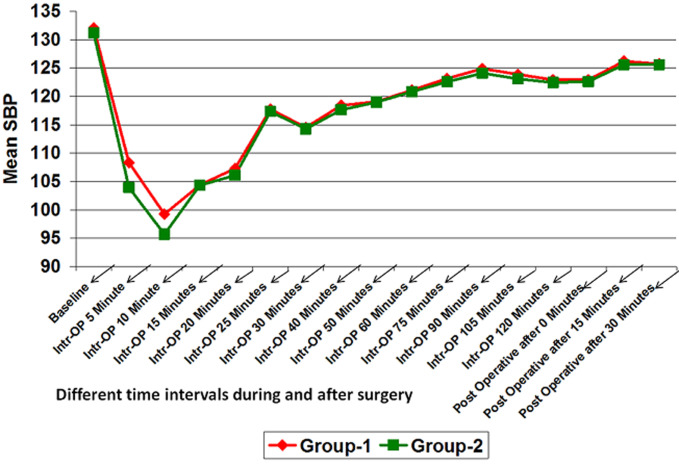
line diagram showing comparison of systolic blood pressure at different time intervals in both the groups

**Figure 3:**
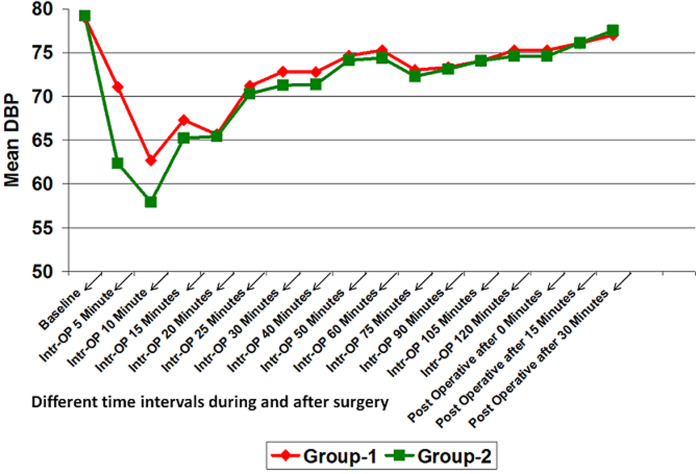
line diagram showing comparison of diastolic blood pressure at different time intervals in both the groups

**Figure 4:**
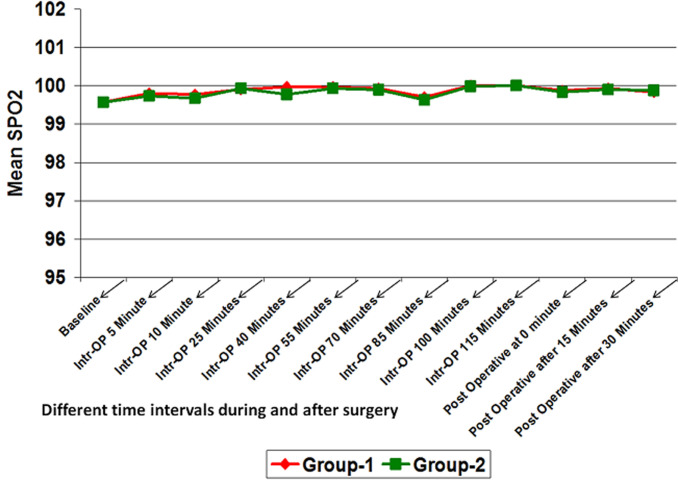
line diagram showing comparison of SPO_2_ at different time intervals in both the groups

**Hemodynamic data:** mean Intra-operative heart rate, SBP and diastolic blood pressure (DBP) in group-2 was lower than that of group-1 at different time intervals. It was significantly lower for intra-operative after 5 minute and 10 minutes. At all other time intervals, although the mean heart rate, SBP and DBP of group-2 was lower than that of group-1 but it was not statistically significant (p>0.05) (Figure 1, Figure 2, Figure 3). However, no significant difference was found in mean SPO_2_ level of group-1 and group-2 at different time intervals (p>0.05) (Figure 4). SPO_2_ did not fall below 98 in any of the patients which suggested that there was no respiratory depression or hypoxia in any patient.

**Peri-operative side effects:** test of proportion showed that proportion of patients with bradycardia and hypotension was significantly higher in group-2 as compared to group-1, whereas the proportion of shivering in group-1 was significantly higher than group-2. There was no significant difference in the proportion of nausea and vomiting between the two groups (Table 4).

**Table 4:** comparison of peri-operative side effects in both the groups

Peri-operative side effect	Group 1 (n=30)	Group 2 (n=30)	Z-value	p-value
Bradycardia	2 (6.6%)	9 (30.0%)	4.27	0.0001
Hypotension	2 (6.6%)	5 (16.5%)	2.19	0.0285
Nausea	3 (10.0%)	2 (6.6%)	0.87	0.38
Vomiting	1 (3.3%)	2 (6.6%)	1.07	0.28
Shivering	6 (20.0%)	1 (3.3%)	3.68	0.0002

## Discussion

In 1891, Quincke demonstrated a safe, predictable means of performing lumbar puncture. The first real spinal anaesthesia was given by August Bier in 1899. He used Quincke´s technique to inject cocaine in order to produce operative anaesthesia [1]. Now-a-days, bupivacaine is a widely used local anaesthetic in spinal anaesthesia. Most commonly used concentration in spinal anaesthesia is 0.5%, which was used in the present study also. However, one of the most common disadvantage of spinal anaesthesia is limited duration of action because of which it may be needed to convert it into general anaesthesia, if surgeries prolonged and hence it cannot be used in prolonged surgeries. To overcome this, different adjuvant drugs were used in past like epinephrine, phenylephrine, adenosine, magnesium sulphate, sodium bicarbonate, neostigmine, but they had their own limitations. Then α-2 agonists like clonidine was introduced in anaesthesia practice. Recently in 1999, dexmedetomidine has been introduced in clinical practice to be used as a better adjuvant to general and regional anaesthesia.

Recent studies have shown the efficacy of intravenous dexmedetomidine in prolonging spinal anaesthesia. Dexmedetomidine is a suitable adjuvant to spinal anaesthesia due to its more selective α-2A receptor agonist activity and by acting at spinal level, laminae VII and VIII of ventral horns. The drug also acts at locus ceruleus and dorsal raphe nucleus to produce sedation and analgesia. This supra spinal action is likely to prolong the spinal anaesthesia after intravenous dexmedetomidine. That is why in the present study IV dexmedetomidine premedication was used on spinal anaesthesia to evaluate its clinical profile [11]. Gupta K *et al*. [12], Reddy VS *et al*. [13], Kaya FN *et al*. [14], Chandrashekharappa K *et al*. [15] and Lee MH *et al*. [16] had used 0.5 µg/kg body weight of dexmedetomidine in their studies, whereas Park SH *et al*. [17] had used 1 µg/kg body weight in their study. Lee MH *et al*. [16] did not find any difference in both the doses, however Park SH *et al*. [17] found prolonged duration of sensory blockade with 1 µg/Kg body weight. In the present study also 1 µg/Kg body weight of dexmedetomidine was used.

In all the above-mentioned studies [13-17], the drug was given as infusion over 10 min as a single bolus dose, prior to SAB similar to the present study. But in the study by Gupta K *et al*. [12], injection dexmedetomidine was given 20 min after SAB. Also, dexmedetomidine infusion was given 5 min prior to SAB in all the studies [12-14,16,17] except in the study by Chandrashekharappa K *et al*. [15], where dexmedetomidine infusion was given 15 min prior to SAB. Peak effect of IV dexmedetomidine infusion was after 5 to 6 min, so SAB was given after 5 min of dexmedetomidine infusion. In the present study single dose of IV infusion was used because literature suggests single dose is sufficient for desired effect on SAB block so it is not necessary to use maintenance infusion, also intrathecal application of dexmedetomidine lacks adequate safety data at the present time [11]. Rapid intravenous administration of dexmedetomidine may cause tachycardia, bradycardia and hypertension [15]. So, in the present study single bolus dose of 1 µg/kg of dexmedetomidine was given over 10 minutes.

**Sensory blockade:** in the present study, the mean onset of sensory blockade observed in group 1 was 5.60 ± 1.22 min and in group 2 was 3.53 ± 1.20 min suggesting statistically significant short onset of sensory blockade in group 2. Thus, suggesting that the premedication with dexmedetomidine had got significant effect on shortening the onset time of sensory blockade. Similarly, pin prick method for assessing the sensory block was used in other studies reported in literature [12-17]. There was significantly higher level of sensory blockade in group-2 than in group-1 in the present study. Similarly, Kaya FN *et al*. [14] and Reddy VS *et al*. [13] also observed the highest level of sensory blockade to be higher in the dexmedetomidine group than control group (p<0.001). Similar findings were observed by Reddy VS *et al*. [13]. In the present study time required for attaining highest level of sensory blockade in group 1 was 7.60 ± 1.43 min and in group 2 was 6.27 ± 1.36 min which is significantly less in group-2 suggesting the effect of dexmedetomidine premedication. None of the other studies have observed this parameter [12-17].

In the present study the mean time required for two dermatomal regression of sensory blockade in group 1 was 86.50 ± 15.93 min and in group 2 was 119.00 ± 13.61 min that suggests that dexmedetomidine premedication significantly prolonged the time for 2 dermatomal regression of sensory blockade. Also, in maximum patients the time required for 2 dermatomal regression was less than 100 min, whereas in group 2, in 24 out of 30 patients the time required for 2 dermatomal regression was more than 100 min. These findings are in accordance with the studies by Gupta K *et al*. [12] (124.35 ± 30.07 min vs 98.54 ± 23.2 min), Reddy VS *et al*. [13] (148.54 ± 20.66 min vs 95.38 ± 17.41 min), Kaya FN *et al*. [14] (145 ± 26 min vs 97.1 ± 26.5 min), Chandrashekharappa K *et al*. [15] (147 ± 14.96 min vs 102 ± 11.71 min), and Lee MH *et al*. [16] (92.5 ± 30.7 min vs 57.6 ± 23.2 min) who found significantly prolonged time for 2 dermatomal regression of sensory blockade in dexmedetomidine group as compared to the control group.

In the present study, the duration of sensory blockade in group 1 was 137.00 ± 14.06 min and in group 2 was 206.33 ± 16.91 min suggestive of significant increase in duration of sensory blockade with premedication of dexmedetomidine. In the present study, all the patients in group 1 showed the duration of sensory blockade upto 200 min whereas in group 2, 20 out of 30 patients showed the duration of sensory blockade more than 200 min. None of the studies had separate observations for sensory regression at L1 level. In the present study the duration of analgesia in group 1 was 176.50 ± 13.84 min and in group 2 was 288.17 ± 19.32 min suggesting that the duration of analgesia was significantly prolonged with premedication of dexmedetomidine. Chandrashekharappa K *et al*. [15] also found that the mean time for first request of post-operative analgesia was significantly prolonged in dexmedetomidine group (240.71 ± 41.13 min) as compared to control group (129.29 ± 17.70 min).

**Motor blockade:** all the other studies [12-17] reported in literature had also used modified bromage scale for assessment of motor blockade. In the present study, early onset of motor blockade was observed in group 2 as compared to the group 1. Similar observations were made in the study done by Chandrashekharappa K *et al*. [15] who found early onset of motor blockade in the dexmedetomidine group. There was no significant difference in the duration of motor blockade. Thus, it suggests that dexmedetomidine premedication is having significant impact on onset of motor blockade but not on total duration of blockade.

**Sedation:** preoperative SED score in both the groups were comparable. In group 1 the score was fairly constant throughout the study period, whereas in group 2 it was significantly higher intraoperatively but its mean value was never more than 3.5 which suggested that the patient was always arousable. This level of sedation is required intraoperatively. In postoperative period it decreased again. Thus, it showed that dexmedetomidine premedication resulted in the intraoperative sedation which is definitely adequate and acceptable.

**Hemodynamic study:** hemodynamically both the groups were stable. Patients showed significantly reduced HR, SBP and DBP at 5 min and 10 min but the change was within clinically safe limit. This is likely to be due to pharmacological action of dexmedetomidine.

**Peri-operative side effects:** many studies have reported significant incidences of bradycardia in the patients varying upto 30% to 40% which required atropine for treatment at some instances during the study. In the present study also the incidence of bradycardia was significantly higher in the group 2 as compared to the group 1. However, these findings are in contrast to the studies done by Reddy VS *et al*. [13], Kaya FN *et al*. [14], Chandrashekharappa K *et al*. [15], Lee MH *et al*. [16], and Annamalai A *et al*. [18] who observed similar incidence of bradycardia among both the groups.

In the present study, the incidence of hypotension was significantly higher in group 2 as compared to group 1, whereas the incidence of shivering was significantly lower in group 2 as compared to group 1. However, the incidence of nausea and vomiting were similar in both the groups. Similar observations were made in the studies by Reddy VS *et al*. [13], Chandrashekharappa K *et al*. [15] who found similar incidence of nausea and vomiting in both the groups.

Observations and result of the present study suggested that there is a definite significant effect of IV infusion of dexmedetomidine on characteristics of sensory blockade after bupivacaine spinal anaesthesia. Onset of motor blockade is also significantly affected but not the duration of blockade. Dexmedetomidine is a selective α-2 agonist. Site of α-2 agonist action is both spinal and supra spinal. The supra spinal action could explain the prolongation of SAB after IV administration of dexmedetomidine [13]. Vasoconstriction action of dexmedetomidine may also contribute to the prolongation of SAB. The effect of dexmedetomidine on the sensory blockade was definitely more than on the motor blockade, which may be because dexmedetomidine produce a greater degree of differential blockade by preferentially blocking the myelinated A (alpha) fibres involved in sensory conduction over unmyelinated c-fibres involved in motor conduction. The postsynaptic activation of α-2 adrenoceptors in the CNS results in decrease in sympathetic activity leading to hypotension and bradychardia. Only few patients may have this effect due to central action of dexmedetomidine, rest of the patients remained hemodynamically stable, it may be because the drug was infused slowly over 10 min. Stimulation of α-2 adrenoreceptors in locus cerularis is responsible for sedative action of dexmedetomidine. This sedative action is also dose dependent, and because of the low dose used in the study, sedation was observed within acceptable limits.

**Limitation:** in the present study, only lower abdominal surgeries were included with the exception of cesarean sections. We recommend future studies with similar study set up to study the effects of IV dexmedetomidine as a premedication in other types of surgeries.

## Conclusion

The present study concludes that slow infusion of IV dexmedetomidine as a premedication significantly prolongs the duration of sensory blockade of 0.5% bupivacaine spinal anaesthesia and hence can be recommended for better and prolonged sensory blockade as well as motor blockade with fairly adequate hemodyamic stability and sedation with minimum incidence of shivering.

### What is known about this topic

- *The highest level of sensory blockade is achieved using IV dexmedetomidine premedication;*
- *Dexmedetomidine premedication significantly prolonged the time for 2 dermatomal regression of sensory blockade*.

### What this study adds

- *Time required for attaining highest level of sensory blockade is significantly less after dexmedetomidine premedication;*
- *There is significant increase in duration of sensory blockade with premedication of dexmedetomidine*.
